# Anti-Ferroelectric Ceramics for High Energy Density Capacitors

**DOI:** 10.3390/ma8125439

**Published:** 2015-11-25

**Authors:** Aditya Chauhan, Satyanarayan Patel, Rahul Vaish, Chris R. Bowen

**Affiliations:** 1 School of Engineering, Indian Institute of Technology Mandi, Mandi 175 001, India; aditya_c@students.iitmandi.ac.in (A.C.); satyanarayan_patel@students.iitmandi.ac.in (S.P.); 2 Department of Mechanical Engineering, University of Bath, Bath BA2 7AY, UK; c.r.bowen@bath.ac.uk

**Keywords:** anti-ferroelectric, energy storage, capacitor, bulk ceramics

## Abstract

With an ever increasing dependence on electrical energy for powering modern equipment and electronics, research is focused on the development of efficient methods for the generation, storage and distribution of electrical power. In this regard, the development of suitable dielectric based solid-state capacitors will play a key role in revolutionizing modern day electronic and electrical devices. Among the popular dielectric materials, anti-ferroelectrics (AFE) display evidence of being a strong contender for future ceramic capacitors. AFE materials possess low dielectric loss, low coercive field, low remnant polarization, high energy density, high material efficiency, and fast discharge rates; all of these characteristics makes AFE materials a lucrative research direction. However, despite the evident advantages, there have only been limited attempts to develop this area. This article attempts to provide a focus to this area by presenting a timely review on the topic, on the relevant scientific advancements that have been made with respect to utilization and development of anti-ferroelectric materials for electric energy storage applications. The article begins with a general introduction discussing the need for high energy density capacitors, the present solutions being used to address this problem, and a brief discussion of various advantages of anti-ferroelectric materials for high energy storage applications. This is followed by a general description of anti-ferroelectricity and important anti-ferroelectric materials. The remainder of the paper is divided into two subsections, the first of which presents various physical routes for enhancing the energy storage density while the latter section describes chemical routes for enhanced storage density. This is followed by conclusions and future prospects and challenges which need to be addressed in this particular field.

## 1. Introduction

As the world actively tries to reduce their dependency on fossil based energy reserves, the successful production, distribution, and storage of electricity will form a cornerstone for the development and growth of society and technology in the coming century. The generation of electricity from renewable and non-conventional resources is being investigated on a large scale and much attention is being paid to the topic of electrochemical energy conversion. However, equal importance is being directed to the development of suitable technologies for efficient storage and distribution of generated electricity. At the forefront of this technology are two main contenders, namely batteries and capacitors. Despite being a relatively well-known concept, the importance of electrical energy storage will continue to be of interest as electricity is the primary means for powering much of our technology. Thus, researchers are working to develop suitable materials with increased energy and power density that are capable of meeting the requirements of modern electric machines and devices [1].

Capacitors as a means for storage of electric energy form an indispensible part of all modern electronic and electrical devices; ranging from electric powered automobiles, drive trains and motors, to mobile communication devices and microwave generation [2,3]. Batteries have a high energy storage density but suffer from a lack of desired power output, while capacitors possess relatively lower energy density but are capable of exhibiting a high power output [2,3]. The main reason behind these differences is the mode of energy storage. Batteries convert electrical energy into chemical energy which is converted back into electrical energy through an electrochemical cell [4,5]. Thus, the rate of up-conversion and re-release of the energy is dictated by the rate of chemical reaction. Capacitors, on the other hand, utilize electrical energy to polarize a dielectric material and thus store energy in the form of localized electric fields/dipole moments existing within the material [2,3]. This energy can be built up over a course of several charging cycles and can be released rapidly to create electrical pulses of required power. This makes for some interesting applications and hence capacitors are employed for a number of devices requiring a high power electric supply [2,3,4,5,6,7]. Regardless of their function, an ideal capacitor is expected to possess a number of basic requirements; a capacitor should possess high power density, high energy density, good discharge efficiency, and low dielectric losses associated with its practical applications [6,7]. Other important requirements include thermal stability and endurance against electrical and mechanical fatigue [6,7]. Therefore, the development of a high energy density electrical capacitor is much sought after as it has the potential to revolutionize the field of power electronics, allowing for rapid miniaturization of modern electronic devices and improving the performance of electric vehicles.

In this regard, much progress has been made in the form of electrochemical supercapacitors [7,8,9,10,11,12]. These are “wet” capacitors which rely on the formation of electrical double layers in a dielectric liquid that can be utilized, when desired, to facilitate electron movement through an external circuit [7,11,12]. This field of research has been the subject of tremendous growth over the last decade and variants have been successfully commercialized for specialized applications. However, the issue of chemical corrosion, fabrication complexity, lack of suitable electrodes, and the danger of spills remains on issue in the current-state-the-art in electrochemical supercapacitors [7,11,12]. Further, their application is limited to devices which allow sufficient space for successful installation and maintenance of such capacitors. As a consequence, this technology is difficult to downscale to suit the continued need for miniaturization in modern electronics. Thus, the need for a suitable material to overcome these shortcomings is still being sought [7,11,12].

A possible route to tackling the issue of providing a power supply to smaller electronic devices lies with the use of solid-state dielectric materials. The maximum energy storage density per unit volume of any dielectric material used for a capacitor application can be estimated using the following relation [13]:

(1) $$U=\frac{1}{2}\cdot \epsilon_{0}\cdot \epsilon_{r}\cdot {E_{b}}^{2}$$

Here, *U* represents the stored energy density per unit volume of the material while *ϵ*_0_, *ϵ_r_* and *E_b_* represent the dielectric permittivity of free space, relative permittivity of the material, and breakdown electric field, respectively. The nature of Equation (1) indicates that materials possessing high dielectric constant (polarizability) and high dielectric breakdown strength are materials of interest for energy storage applications [13]. Furthermore, the energy density of a material increases with the square of the electric field as opposed to linear increase with respect to dielectric constant [13]. Hence, it would appear to be highly beneficial to have a material which can sustain large electric field intensities so as to improve its energy storage characteristics. In this regard, the use of glassy materials as a dielectric medium for capacitor applications is of interest since they possess high dielectric breakdown strengths (~10^9^ V/m) [14,15,16,17]. They are also relatively easy to process in a required shape, along with low fabrication costs and good thermal stability [17]. All these factors indicate that glasses are promising materials for electrical energy storage. However, this particular approach to the storage of electrical energy at higher electric fields has some disadvantages; when high electric fields are utilized for energy storage applications the device requires special insulating and shielding materials to protect nearby components from electrical discharge [18,19]. This inevitably raises the overall equipment cost. Secondly, special wiring and installation is required to allow safe charging and discharging of the electrical capacitor when working at high operating voltages. Thirdly, at higher electric fields there is an increased risk of dielectric breakdown and this raises several safety concerns [20]. “Theoretically” for the same amount of voltage, a material with high dielectric constant will possess more energy density and conversely, for the same energy density lower dielectric constant would require a higher electric potential. Anti-ferroelectrics (AFE) materials possess significantly higher dielectric constants than glassy materials which are linear dielectrics. Therefore, the same amount of energy can be stored at a lower value of electric field making it a safer option. A higher voltage runs the risk of corona leakage, electrical aging/fatigue, catastrophic dielectric breakdown and insulation failure. Hence, an anti-ferroelectric (AFE) material with similar energy density is safer for energy storage than linear dielectrics. Furthermore, since glass possesses a poor level of polarizability, the application of a high electric field (in the order of ~10–12 MV/cm) is required to store utilizable energy [21]. An exception to this rule is observed with nano-structured TiO_2_ which have been reported for exceptional energy storage characteristics possessing a dielectric constant of 110 with a dielectric breakdown strength of 1400 kV/cm. These figures’ combined yield an effective energy storage density of 14 J/cm^3^ [22]. However, in order to realize this high energy density, an electric field of substantially high intensity is required which again translates back to the original plight of high electric potential. It is for similar reasons that the use of (ferroelectric/dielectric) polymers as capacitor materials have not gained much confidence [23,24,25]. In addition, polymers have relatively poor thermal stability compared to glasses even though ferroelectric co-polymers have been reported to possess larger dielectric constants in comparison to glasses [25,26,27,28]. The third possible class of material in this application is dielectric and ferroelectric thin films [29,30]. Due to their low defect concentration, thin films are capable of withstanding high electric fields [31] and hence possess exceptionally large volume specific energy storage density [32]. However, the absolute energy stored is low for practical purposes and hence such devices are used in configurations having multiple storage layers, which is essentially a device problem. Therefore, thin films have been excluded from discussion in this article. Current research is focused on development of materials which are capable of achieving high energy storage density at relatively low electric fields. In this regard, several other categories of dielectric materials are being extensively explored.

**Figure 1:**
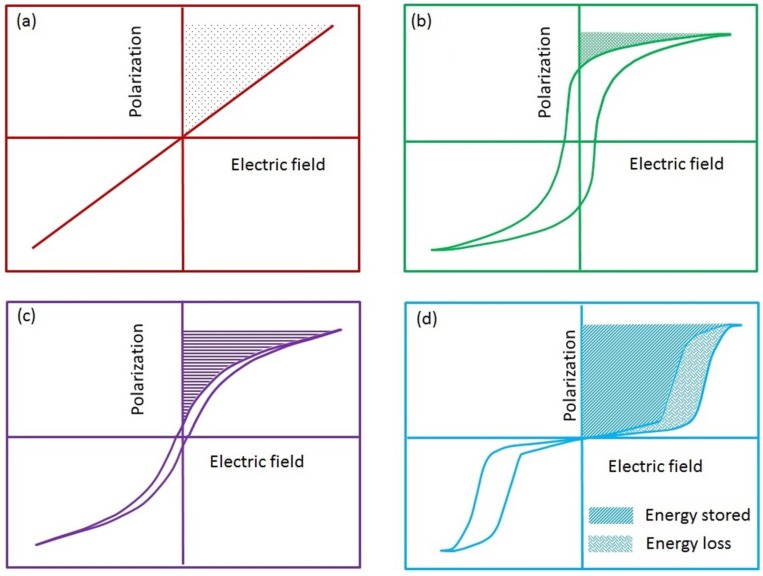
Figure represents the typical polarization *versus* electric field (*P*-*E*) hysteresis loops and energy storage characteristics of the four classes of solid dielectric materials namely (**a**) linear; (**b**) ferroelectric; (**c**) relaxor ferroelectric; (**d**) anti-ferroelectric (demonstration only; not to scale).

The majority of materials used for fabrication of electrical capacitors consist of linear dielectrics [33], ferroelectrics [34,35], relaxor ferroelectrics [32,36], and anti-ferroelectrics. Figure 1 provides a graphical representation of each of the material classes and their energy storage characteristics as indicated on a polarization *versus* electric field (*P*-*E*) hysteresis loop [37]. The hatched area represents dischargeable energy while the area within the hysteresis loop represents dielectric losses. Further, a ratio of these energies can be used to determine the energy storage efficiency of the material. Discussion for all these factors will be provided in detail in this article. Glass is a good example of linear dielectric which has already been discussed earlier (Figure 1a). Linear dielectrics are often characterized by their low dielectric constant and high dielectric breakdown strength. In contrast, the second most popular choice for capacitor applications has been ferroelectric materials which possess a large dielectric constant but suffer from lower dielectric breakdown strength. Additionally, ferroelectric materials suffer from dielectric losses during charging and discharging cycles as a result of the ferroelectric hysteresis (Figure 1b). Nevertheless, with modern advances in the field of materials chemistry and fabrication techniques it is now possible to tune the dielectric response of such materials [38,39]. In addition, the energy densities compared to high voltage linear dielectrics have been reported [38,39]. Relaxor ferroelectrics are considered to be dipolar glasses displaying a lack of long-range order with respect to ferroelectric domains. As a consequence they possess almost negligible remnant polarization and coercive electric field while also displaying a lack of conceivable ferroelectric hysteresis (Figure 1c). Thus, they are gaining popularity over conventional ferroelectric materials as a suitable candidate for capacitor applications [40,41]. However, material systems which display relaxor characteristics are relatively rare [41] and are mostly lead-based, for example *x*Pb(Mg_1/3_Nb_2/3_)O_3_-(1 − *x*)PbTiO_3_. Hence, due to growing environmental concern towards reducing the use of lead in ferroelectric materials, a more suitable material is being sought.

Anti-ferroelectric materials possess relatively larger energy storage density, have lower values of remnant polarization and coercive electric field and faster discharge rates for dissipating stored electrical energy, due to ferroelectric to anti-ferroelectric phase transition [42,43]; see Figure 1d. Due to the lack of ferroelectric domains at low electric field, AFE materials do not possess any considerable hysteresis and have low remnant polarization, coercive electric field, and dielectric loss. However, at higher electric fields, the anti-parallel dipoles are aligned to form a ferroelectric phase. Thus, these materials are capable of storing considerable energy at higher material efficiencies and exhibit faster discharge rates. Appropriate details for the same have been provided in subsequent sections. Thus, upon considering the associated facts with each material family, a consensus can be achieved that anti-ferroelectric materials are a natural choice as potential candidates for electrical energy storage capacitors.

Anti-ferroelectric ceramics in their innate state are characterized by the presence of anti-parallel dipolar alignment [37,44,45]. This arrangement of polar moment in the material creates a complete absence of any observable ferroelectric, piezoelectric, and pyroelectric properties. However, under the application of a sufficiently high intensity electric field, the dipoles rotate to align in the direction of the applied external field [38,39]. This is followed by a first order reversible anti-ferroelectric to ferroelectric phase transformation. The magnitude of the electric field required for the phase change depends on material composition and external parameters such as temperature and stress. Thus, at high fields, the anti-ferroelectric material behaves as a ferroelectric material. Since the transformation is reversible, the ferroelectric phase reverts back to its original anti-ferroelectric form when the applied electric field is reduced below a certain critical value [43,46]. The low hysteresis losses coupled with the reversible phase transformation have been associated with high power and energy density in anti-ferroelectric materials [37,47,48]. Furthermore, the loss of ferroelectric characteristics at low electric field is also responsible for a fast discharge rate and high discharge efficiency in these materials. Thus, anti-ferroelectric compositions have the potential to become high performing contenders in the field of commercial capacitor applications.

Excellent reviews are present in the literature dealing with development and applications of a wide range of anti-ferroelectric materials [49,50]. However, little or no attention has been paid to the most promising attribute of this material class: high energy storage capacitors. Further, the amount of research conducted in this direction is also limited due to a lack of variety in materials with anti-ferroelectric properties [51,52]. However, with advancements in the field of fabrication and characterization techniques and the discovery of novel lead-free ferroelectric materials, research in this field can be reignited [53]. We have attempted to undertake this review article and provide the necessary spark by presenting a timely assessment on the subject. This article aims to review and highlight the relevant scientific advancements that have been made with respect to utilization and development of anti-ferroelectric materials for electric energy storage applications. The structure of this paper is as follows: the article begins with a general introduction discussing the need for high energy density capacitors, the present solutions being used to address this problem and a brief discussion of various advantages of anti-ferroelectric materials for high energy storage applications. This is followed by a general description of anti-ferroelectricity and important anti-ferroelectric materials. The remainder of the paper is divided into two subsections, the first of which presents various physical routes for enhancing energy storage density while the latter section describes how chemical routes can be used to accomplish the same. This is followed by conclusions and future prospects and challenges which need to be addressed in this particular field.

## 2. Anti-Ferroelectricity and Associated Materials

**Figure 2:**
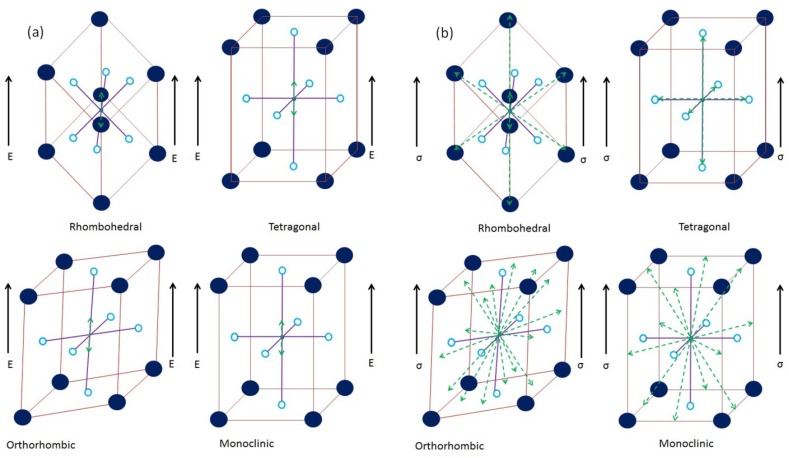
Graphical representation of the mechanism and various switching directions associated with different crystallographic structures under (**a**) ferroelectric; (**b**) ferroelastic domain rotation, respectively.

AFE materials are similar to ferroelectric materials and largely belong to the perovskite family represented by the nominal chemical formula of ABO_3_; here A represents an alkali or alkaline earth metal while B belongs to the transition group elements [54,55]. This structure gives rise to the BO_6_ octahedra where the transition group element is surrounded by six oxygen atoms [54,55,56]. When the atom is eccentrically placed, it causes the separation of the charge centers, giving rise to a permanent dipole vector within the crystal. Large numbers of such dipoles are aligned together in a ferroelectric material giving rise to ferroelectric domains [54,55]. In a bulk material, many such domains are present, arranged in random directions leading to an electrically neutral material [54,55,56]. However, the application of an external electric field can align dipoles in a predetermined manner, as shown in Figure 2 [56]. In AFE materials, adjacent layers of octahedra have dipoles aligned in opposite directions (anti-parallel), in the absence of electric field [50]. Thus, even at microscopic level they appear to lack any domain structure and display a complete lack of electrical functionality. However, when an external electric field beyond a certain threshold is applied to AFE materials, the dipoles are rotated and give rise to a domain structure, as well as ferroelectric behavior [43]. This is evident from the double hysteresis loop (Figure 1d) which is often used to identify the AFE materials. Thus, an AFE phase can be converted into a ferroelectric phase upon the application of electric field of suitable strength. Consequently, these materials lack a major part of low-field hysteresis which is associated with conventional FE materials and thus possess lower dielectric losses which greatly increases the energy storage efficiency. Furthermore, the AFE to FE transition is accompanied by a volumetric expansion in both longitudinal and transverse directions and has been reported to be sensitive to both applied temperature and stress [43,46]. Table 1 provides a list of the known bulk AFE materials and their associated properties as reported in the literature. While it is not comprehensive, care has been taken to acknowledge any and all relevant results reported in the literature with regards to employment of AFE materials for energy storage applications. Another important point to be mentioned here is that the list provides energy densities which were obtained for a very specific material geometry and processing parameters. Hence, it is entirely possible to obtain values different than those reported here, based on the material’s shape (area/thickness), size, and processing.

**Table 1:** Important bulk anti-ferroelectric materials, with various physical/chemical modifications for enhancing energy storage density, as reported in literature.

Material Name	Physical/Chemical Modifications	Energy Density (J/cm^3^)	Electric Field (kV/cm)	Reference
(Pb_0.97_La_0.02_)(Zr_0.97_Ti_0.03_)O_3_	3 wt % glass (PbO-B_2_O_3_-SiO_2_-ZnO)	3.1	581	[57]
(Pb_0.97_La_0.02_)(Zr_0.97_Ti_0.03_)O_3_	without glass	1.4	581	[57]
Pb_0.99_Nb_0.02_[(Zr_0.57_Sn_0.43_)_1 − *y*_Ti_*y*_]_0.98_O_3_	with half electrode	1.30	70	[47]
Pb_0.99_Nb_0.02_[(Zr_0.57_Sn_0.43_)_1 − *y*_Ti_*y*_]_0.98_O_3_	with full electrode	1.19	70	[47]
(Pb_0.94 −*x*_La_0.04_Ba_*x*_)[(Zr_0.60_Sn_0.40_)_0.841_Ti_0.16_]O_3_	*x* = 0 (0 MPa)	0.35	30	[58]
(Pb_0.94 − *x*_La_0.04_Ba_*x*_)[(Zr_0.60_Sn_0.40_)_0.841_Ti_0.16_]O_3_	*x* = 0.02 (20 MPa)	0.38	30	[58]
Pb_0.99_Nb_0.02_[(Zr_0.60_Sn_0.40_)_0.95_Ti_0.05_]O_3_	-	0.62	56	[59]
Pb_0.97_La_0.02_(Zr_0.56_Sn_0.35_Ti_0.09_)O_3_	4 wt % glass (CdO-Bi_2_O_3_-PbO-ZnO-Al_2_O_3_-B_2_O_3_-SiO_2_)	3.3	150	[60]
Pb_0.97_La_0.02_(Zr_0.56_Sn_0.35_Ti_0.09_)O_3_	without glass	1.9	110	[60]
(Pb0_:97_La_0:02_)(Zr_0:92_Sn_0:05_Ti_0:03_)O_3_	3% glass (0.8PbO-0.2B_2_O_3_)	7.4	475	[61]
(Pb0_:97_La_0:02_)(Zr_0:92_Sn_0:05_Ti_0:03_)O_3_	without glass	4.5	320	[61]
Pb_0.97_La_0.02_(Zr_0.95_Ti_0.05_)O_3_	-	12.4	1120	[62]
(Pb_0.85_Ba_0.08_Sr_0.03_La_0.03_)(Zr_0.74_Sn_0.22_Ti_0.04_)	-	1.2	100	[63]
[(Bi_1/2_Na_1/2_)_0.94_Ba_0.06_]La_0.8_Zr_0.2_TiO_3_	-	1.58	85	[64]
(Ba_0.1_La_0.02_)(Zr_0.675_Sn_0.275_Ti_0.05_)O_3_	-	2.05	70	[65]
0.75(_0.80_Bi_1/2_Na_1/2_TiO_3_-0.20Bi_1/2_K_1/2_TiO_3_)-0.25SrTiO_3_	-	0.84	100	[66]
Pb_0.97_La_0.02_(Zr_0.50_Sn_0.45_Ti_0.05_)O_3_	-	5.6	400	[67]
(Pb_0.858_Ba_0.1_La_0.02_Y_0.008_)(Zr_0.65_Sn_0.3_Ti_0.05_)O_3_-(Pb_0.97_La_0.02_)(Zr_0.9_Sn_0.05_Ti_0.05_)O_3_	-	4.65	200	[68]
0.89Bi_0.5_Na_0.5_TiO_3_-0.06BaTiO_3_-0.05K_0.5_Na_0.5_NbO_3_	double stage sintering	0.90	100	[69]
(Pb_0.858_Ba_0.1_La_0.02_Y_0.008_)(Zr_0.65_Sn_0.3_Ti_0.05_)O_3_-(Pb_0.97_La_0.02_)(Zr_0.9_Sn_0.05_Ti_0.05_)O_3_	spark plasma sintering	6.40	275	[70]
(Pb_0.858_Ba_0.1_La_0.02_Y_0.008_)(Zr_0.65_Sn_0.3_Ti_0.05_)O_3_-(Pb_0.97_La_0.02_)(Zr_0.9_Sn_0.05_Ti_0.05_)O_3_	conventional sintering	4.65	200	[70]
(Na_1 − *x*_Ca_*x*_)(Nb_1 − *x*_Zr_*x*_)O_3_ *x* = 0.04	conventional sintering	0.91	130	[71]
(Pb_0.92_La_0.04_Ba_0.02_)[(Zr_0.60_Sn_0.40_)_0.84_Ti_0.16_]O_3_	at 90 MPa	0.91	60	[43]
0.91(Bi_0.5_Na_0.5_)TiO_3_-0.07BaTiO_3_-0.02(K_0.5_Na_0.5_)NbO_3_	at 100 MPa	0.387	60	[37]
(Pb_0.96_La_0.04_)(Zr_0.90_Ti_0.10_)O_3_	at 100 MPa	0.698	60	[43]

Due to their high field transformation, low hysteresis, low remnant polarization and high energy storage density, AFE materials have been proposed as leading contenders for solid-state capacitor applications [50,51,52,66,72]. However, since the materials typically belong to the perovskite ceramic family they suffer from low dielectric breakdown strength [73,74]. This serves to limit the maximum amount of energy that can be stored in these materials which ultimately falls behind that reported for linear dielectrics. Hence, efforts have been made to increase the energy storage density in AFE. This approach can be subdivided into two main techniques namely physical and chemical routes which attempt to reduce hysteresis, increase the dielectric constant, or enhance the dielectric breakdown strength of the AFE. The following sections will discuss in detail how each of these methods is accomplished and the relevant studies which have reportedly employed these techniques to enhance the energy storage density in AFE materials.

## 3. Enhancing Energy Storage Capacity in Anti-Ferroelectric Materials: Physical Routes

As explained previously, Equation (1) can be used to evaluate the energy storage density of a dielectric material. However, another popular method for determining the energy storage density in ferroelectric, relaxor ferroelectric, and anti-ferroelectric materials is to numerically integrate the area under the polarization *versus* electric field (*P*-*E*) hysteresis curves (as in Figure 1). The numerical representation for this is given as [43,75]:

(2) U=∮E·dP

Here *E* is applied electric field and *P* is polarisation. The expression in Equation (2) can be used to determine the energy density of most ferroelectric materials at their saturation electric field values. Furthermore, since ferroelectric materials are prone to hysteresis losses, another important factor known as material efficiency needs to be determined according to the following relation [37]:

(3) $$\eta =\frac{U}{U+U_{1}}$$

Here the symbols η and *U*_1_ represent the material efficiency and dielectric losses respectively. It can be clearly understood from Equation (3) that if dielectric losses can be reduced, it leads to improved energy storage characteristics. It has already been established in the literature that an AFE to FE transition is sensitive to both ambient temperature and applied pre-stress to the material [76,77,78]. Hence, the physical methods are concentrated on how to utilize these effects in order to delay the transition, so as to increase the energy density or reduce hysteresis losses and thereby increase material efficiency. These have been discussed in detail as follows.

### 3.1. Compressive Pre-Stresses

When an electric field is applied to an AFE material it continues to energize the anti-parallel dipole alignment until the threshold intensity is reached, at which the FE characteristic is initiated. This is accompanied by a large volumetric expansion and corresponding strain [79,80]. The process is reversed when the applied electric field is lowered below a certain critical point. This difference in forward and backward switching electric fields is known as Δ*E* [79,80]. This phenomenon is similar to the ferroelectric domain rotation as observed in conventional FE materials. Therefore, assuming AFE to be initial phase and FE to be final phase for forward process, the minimum energy required to initiate domain formation can be dictated using the Helmholtz free energy density (*A*) for ferroelectric materials as follows [81]:

(4) dA=σdϵ+EdD−SdT

Here, σ, ϵ, *E*, *D*, *T* and *S* denote stress, strain, electric field intensity, electric displacement, temperature, and entropy, respectively. From Equation (4) it can be deduced that the equilibrium can be shifted in the favor of a preferred state by a suitable application of stress, electric field or temperature. Therefore, Equation (4) can be rewritten to represent a phase change as follows [82]:

(5) $${dA}^{\alpha \to \beta }={\sigma d\epsilon }^{\alpha \to \beta }+EdD^{\alpha \to \beta }+TdS^{\alpha \to \beta }$$

Here, α and β indicate the initial (AFE) and final (FE) phases of material. This effect has been reported to drive a phase change in a large number of studies [46,59,83,84,85,86,87]. It can also be inferred from Equations (4) and (5) that if *d*ϵ ≠ 0, an externally applied mechanical stress will influence the phase transformation. The nature of influence of compressive stresses on any structural transition depends on *d*ϵ. As is the case of AFE to FE phase change, it is accompanied by volumetric expansion in both lateral and axial directions. Thus, the application of a compressive stress or mechanical confinement to the material will shift the value of electric field required to drive the transition to a higher magnitude [58,78]. This phenomenon can be explained on the basis of the competing nature of mechanical and electrical domain switching modes. In its innate state, a bulk ferroelectric ceramic consists of randomly aligned domains. Thus, the material as a whole does not exhibit any spontaneous polarization or ferroelectric activity. However, when the same material is subjected to an electric field of increasing magnitude, domain realignment is initiated. Initially, minor domain wall motion is observed which favors the growth of preferentially aligned domains [43,46,86,87]. However, upon being subjected to higher magnitude of electric field, the domains themselves are rotated in the direction of the applied electric field. This phenomenon is known as ferroelectric domain switching and is generally associated with 180° domain rotation [37,43,46,58,62,78]. Conversely, when a poled or activated, ferroelectric material is subjected to compressive stress, the domains realign to face away from the direction of stress applied; termed ferroelastic domain switching [43,86,87]. The movement of the domains is such that it minimizes structural energy to accommodate the additional strain being generated in the material. Ferroelastic domain rotations are inherently non-180° in nature and the switching direction has been observed to be dependent on the crystallographic structure of the material [37,43,46,58,62,78]. A graphical description of the ferroelectric and ferroelastic domain switching (directions) associated with different crystallographic structures is shown in Figure 2. It can be clearly seen from Figure 2 that each crystal structure has several possible switching sites available to it under electric field or stress. The application of an electric field generally forces the movement of a charge center in such a manner that it minimizes the electrical strain of the material, through the formation of electrical dipoles [37,43,46,58,62,78]. This is generally accomplished by displacing the charge centers along the direction of applied electric field (180° switching). Conversely, when such a poled material is subjected to the application of a compressive stress the charge centers are displaced in such a manner that it minimizes the strain energy of the system. This is accomplished by shifting the “mobile” charge center to one of the available side sites (non-180° switching) [37,43,46,58,62,78].

**Figure 3:**
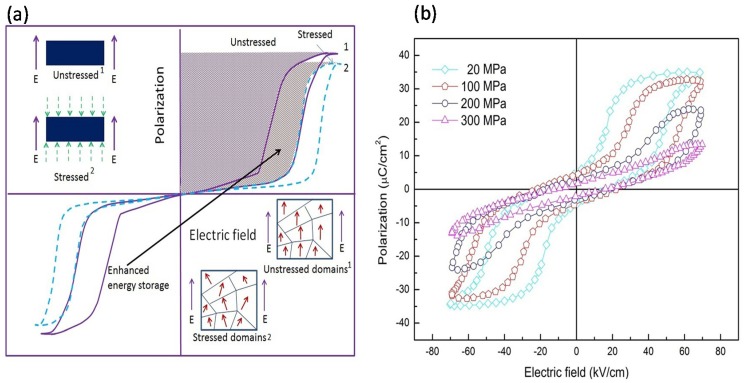
Graphical representation for the technique of physical confinement to increase the energy storage density in anti-ferroelectric materials. (**a**) Displays how the hysteresis loop changes to yield enhanced recoverable energy under uniaxial compressive stress while (**b**) displays *P*-*E* hysteresis loops for (Pb_0.96_La_0.04_)(Zr_0.90_Ti_0.10_)O_3_ material under compressive loading [88].

Thus, when a material is subjected to a combined electro-mechanical loading, the domain alignment is decided by the equilibrium value of the competing fields. It is possible to utilize this phenomenon to also create stress-induced domain pinning in AFE materials [58,78]. Since hysteresis is the equivalent energy utilized by the electric field in rotating the dipoles, domain pinning counters this effect directly. With increasing magnitude of applied compressive stress, a growing number of dipoles are “pinned” to their position. Consequently, the switchable part of polarization is reduced and this is manifested in the form of reduced hysteresis. The phenomenon has been demonstrated using Figure 3 where Figure 3a shows the two states of ferroelectric hysteresis observed in AFE materials in the absence and presence of a compressive stress. It can be observed that the application of compressive stress leads to a loss of saturation polarization with a simultaneous reduction of hysteresis. The inset shows the corresponding nature of field induced ferroelectric domains. Figure 3b shows *P*-*E* hysteresis curves for (Pb_0.96_La_0.04_)(Zr_0.90_Ti_0.10_)O_3_ AFE material under various compressive stresses [88]. However, it is to be noted that ferroelectric and ferroelastic switching act to counter each other.

Thus, if the magnitude of one of the fields is increased beyond a certain critical value it will completely override the effect of other. In such a situation, no phase change can be initiated and thus material performance begins to degrade.

In a study reported by Xu *et al.* it was observed that in bulk AFE ceramics having a nominal composition of (Pb_0.94 - *x*_La_0.04_Ba_*x*_)[(Zr_0.60_Sn_0.40_)_0.84_Ti_0.16_]O_3_, the energy storage density could be increased through mechanical confinement in (*x* =) 0.02 and 0.04 compositions [58]. Barium modification of the material was seen to reduce the domain size and make the pre-stress less effective in preventing domain polarization alignment. However, for enhancing the energy density, an optimum value of compressive stress is required, in accordance with Equation (5). This was also observed for composition of *x* = 0.02 where the maximum energy storage density was observed at 70 MPa (~14.8% increase) and any further increase in pre-stress led to a decrease in energy storage density.

Our group has attempted to enhance the energy storage density in multiple AFE ceramics through the application of compressive pre-stress. We undertook systematic investigations to examine the effect of directional confinement on the energy storage density of 0.91(Bi_0.5_Na_0.5_)TiO_3_-0.07BaTiO_3_-0.02(K_0.5_Na_0.5_)NbO_3_ bulk AFE ceramic [37]. Hysteresis behavior was analyzed as a function of varying levels of compressive stress and operational temperature. It was observed that a peak energy density of 0.387 J/cm^3^ was obtained at 100 MPa applied stress (25 °C), while a maximum energy density of 0.568 J/cm^3^ was obtained for the same stress at 80 °C. These values are indicative of a significant, 25% and 84%, improvement in the value of stored energy compared to an unloaded material. A further study [37] clearly revealed that under the application of a compressive stress, the material performance increased rather than decreased, as the operating temperature was increased. The study was also extended to analyze the discharge efficiency under various loading conditions. The material’s discharge efficiency was also improved considerably owing to thermal and mechanical fields. The unloaded sample exhibited an efficiency of 37%, which was later improved to a value of 49% for 250 MPa at room temperature and 72% for 100 MPa at 80 °C. A peak efficiency of 90.42% was observed for measurement conditions of 140 °C and 250 MPa applied stress [37].

The second analysis [43] was based on bulk (Pb_1−*x*_La_x_)(Zr_0.90_Ti_0.10_)_1–*x*/4_O_3_ AFE ceramics. Mechanical confinement was observed to provide an increase in energy storage density and efficiency by approximately 38% and 25%, respectively, for the *x* = 4 composition [43]. The highest recoverable energy density of 0.698 J/cm^3^ was achieved under a compressive stress of 100 MPa and electric field of 60 kV/cm. In this composition, the storage density could be improved from 0.536 J/cm^3^ to 0.704 J/cm^3^ for a temperature increase of 25 to 120 °C, respectively. Similarly, the storage efficiency also increased from 41% to 67% with an increase in temperature from 25 to 120 °C. This is due to fact that when the ferroelectric/anti-ferroelectric material is heated, it relaxes and disorients the domains. This reduces the reversible part of ferroelectric switching, which ultimately decreases the area under the hysteresis loop. Therefore, the energy loss of the material decreases while simultaneously increasing the storage density and efficiency. In contrast, a sufficiently high temperature also reduces saturation polarization and, in addition, temperature also affects the dielectric constant of the material. In summary, the application of elevated temperature (120 °C) can increase approximately 31% and 25% storage density and efficiency, respectively, as compared to normal storage density and efficiency (25 °C) in *x* = 4 composition.

### 3.2. Self Clamping Using Electrodes

**Figure 4:**
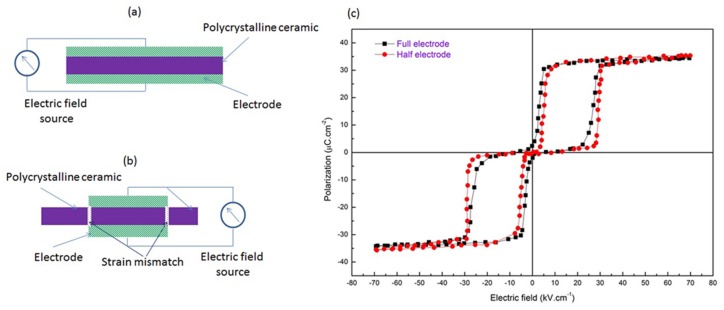
Graphical representation of anti-ferroelectric ceramics’ cross-sectional view of (**a**) completely electroded ceramic; (**b**) half electroded ceramic (green is electrode (hatched), purple is ceramic); and (**c**) *P*-*E* hysteresis loops for of Pb_0.99_Nb_0.02_[(Zr_0.57_Sn_0.43_)_1−*y*_Ti_*y*_]_0.98_O_3_ material under induced self-confinement. Data adapted from [47].

It is often difficult to create a pre-stress in ceramic capacitors, especially when they form an integral part of an integrated circuit. From the literature it is evident that a compressive stress can be utilized to greatly improve the performance of AFE based capacitors [37,43,58,76,78,89]. Thus, in order to overcome the limitation of applying pre-stresses an alternative method has been proposed, known as self-confinement, using patterned electrodes. The mechanism has been explained using Figure 4. Figure 4a,b describe the symmetrical arrangements of full and half electrodes in a circular parallel plate ceramic capacitor.

When an electric field is applied to this arrangement, the fully electroded area undergoes volumetric expansion as a consequence of the AFE to FE phase transformation. However, un-electroded section remains unaltered, leading to a strain mismatch between different volumes of the ceramic (see Figure 4b); which is similar to inducing a compressive pre-stress within the material. One of the notable attempts in this regard is that reported by Young *et al.* [47] who used a concentric patterned electrode to induce an effective compressive stress of 30 MPa in the electroded portion of the disk; this helped to increase the energy storage density. The study was supported by a phase-field model and was carried out using bulk samples of Pb_0.99_Nb_0.02_[(Zr_0.57_Sn_0.43_)_1−*y*_Ti_*y*_]_0.98_O_3_ AFE ceramics, the *P*-*E* loops are shown in Figure 4c [47]. When compared to symmetrically coated samples, an increase of ~10% in energy density was observed from 1.19 to 1.30 J/cm^3^ for *y* = 0.06 samples.

## 4. Enhancing Energy Storage Capacity in Anti-Ferroelectric Materials: Chemical Routes

### 4.1. Glass Incorporation and Internal Clamping

**Figure 5:**
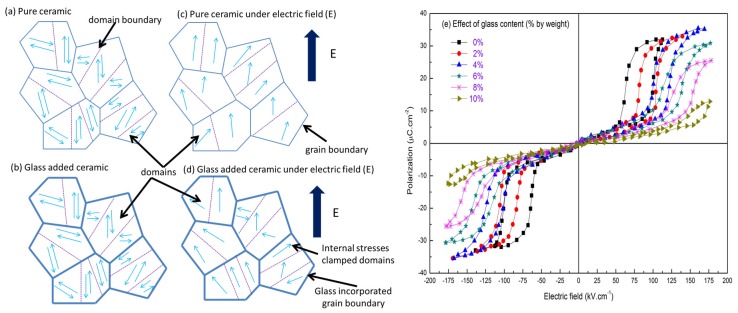
The effect of internal clamping at a microscopic level due to glass incorporation, in anti-ferroelectric material for (**a**) pure ceramic; (**b**) ceramic with glass incorporation; (**c**) pure ceramic under electric field; and (**d**) glass-ceramic composite under electric field and their domain behavior; (**e**) *P*-*E* hysteresis loops for Pb_0.97_La_0.02_(Zr_0.56_Sn_0.35_Ti_0.09_)O_3_ glass ceramic composite [60].

Another effective method to increase the energy storage density within AFE ceramics is to incorporate chemically inert glass into the ceramic matrix. Glasses have been added in ferroelectric ceramics in order to reduce the sintering temperature using a viscous sintering approach [90,91]. Glasses have a high electric breakdown strength [21,92,93,94] and careful selection and addition of glasses in ferroelectric ceramics can enhance their energy storage properties. This technique is especially effective if the glass phase resides at the grain boundaries. Grain boundaries in a bulk ceramic are physical discontinuities and act as points of stress concentration owing to the high defect concentration existing on the surface of the grains. Most dielectric failures in ferroelectrics are an indirect consequence of defect concentration at grain boundaries [95] and if the glass can fill the grain boundary discontinuity, it can enhance the electric breakdown strength. However, glass additions can also drastically affect shape, size and growth rate of grains in the ceramics which are responsible for controlling various physical properties [96]. The addition of a glass phase can also produce the required mechanical confinement. Figure 5 shows the polarization behavior of a ferroelectric ceramic (Figure 5a,c) and ferroelectric with added glass (Figure 5b,d) under an electric field. In this situation, domains are also oriented in the direction of applied field. However, they possess some degree of independent orientation which depends on glass content and crystal structure, such as rhombohedral/tetragonal, of the ceramic composite. Internal clamping of domains can be induced due to the presence of glass. This is due to the fact that under an applied electric field, AFE ceramics are strained due to a FE phase transformation. However, glass is non-ferroelectric and remains uneffected and an internal stress is generated between the glass and ceramics phases. Hence, it can be concluded that the addition of a glass leads to ferroelastic domain switching away from the applied electric field. The orientation is in the energetically preferred direction(s) and ultimately reduces the number of switching sites available which helps reduce dielectric losses. Figure 5e shows a typical *P*-*E* loop for CdO-Bi_2_O_3_-PbO-ZnO-A_l2_O_3_-B_2_O_3_-SiO_2_ glass addition and energy storage performance of Pb_0.97_La_0.02_(Zr_0.56_Sn_0.35_Ti_0.09_)O_3_ [60]. The glass was added to serve the dual purpose of lowering the sintering temperature and improving the dielectric breakdown strength of the fabricated samples. However, it was noted that with increasing glass content the electric field required for AFE to FE phase transformation also increased, while Δ*E* decreased simultaneously; this may be a direct consequence of the internal clamping phenomenon. Glass additions were used to lower the sintering temperature from ~1300 to 1050 °C where high densities (>93%) were achieved due to viscous sintering and a reduction of grain size. Furthermore, the incorporation of glass was reported to yield several direct benefits such as decreasing dielectric loss and improving discharge efficiency. The *P*-*E* loops of glass containing ceramics also showed a progressive lack of squareness and slanted loops were obtained. It was observed that the sample containing 4 wt % glass and sintered at 1130 °C displayed a large energy storage density of 3.3 J/cm^3^, with a high discharge efficiency of 80%.

In other work, glass additions have also been investigated by Chen *et al.* who fabricated 50 μm-thick (Pb_0.97_La_0.02_)(Zr_0.92_Sn_0.05_Ti_0.03_)O_3_ antiferroelectric (AFE) thick films with different amounts of 0.8PbO-0.2B_2_O_3_ glass additions [61]. The films were fabricated by a screen printing method on an alumina substrate with a platinum electrode. It was reported that the incorporation of a glass phase helped to enhance the dielectric breakdown strength of the thick films which in turn yielded higher energy storage densities. Upon incorporation of 3 wt % glass, the films were able to withstand an electric field intensity of 475 kV/m with a reported energy density of 7.4 J/cm^3^ and a discharge efficiency of 55%.

Furthermore, the saturation polarization was improved with up to 3 wt % glass addition with a pristine film having a maximum polarization of 26.2 μC/cm^2^ while a 3 wt % glass film having a higher polarization of 34.8 μC/cm^2^. Hao *et al.* have reported the effect of glass addition on microstructure and energy storage characteristics on (Pb_0.97_La_0.02_)(Zr_0.97_Ti_0.03_)O_3_ AFE thick films [57]. They used PbO-B_2_O_3_-SiO_2_-ZnO glass ranging from 0 to 5 wt % and fabricated the composites using a screen printing method. The addition of glass was reported to favor the formation of a denser microstructure. Samples containing 3 wt % glass was reported to possess the highest average breakdown strength of 581 kV/cm, which is 140% larger than pristine films. The dielectric constant of the composite was also improved compared to pure perovskite phase and an energy density of 3.1 J/cm^3^ was reported, which is more than twice that of a pure ceramic sample. However, the discharge efficiency was observed to decrease with increasing electric field intensity and dropped from a maximum value of 72% at 258 kV/cm to 35% at 581 kV/cm. Thus, the application of a high intensity electric field is not necessarily advantageous for obtaining improved energy density. Glass addition was also reported to impart a good thermal stability to the ceramics within the temperature range of 20 to 120 °C which is also an additional benefit of glass incorporation within the ceramic.

### 4.2. Doping and Chemical Modifications

Tailoring of the material chemistry in the form of solid-solutions, heterovalent and isovalent doping has been extensively reported in the literature to tune the response of any functional material [97,98,99,100,101,102]. The same has also been proposed for enhancing the energy storage characteristics in various AFE materials, where doping has been proven highly effective. In a study by Jiang *et al.* they investigated the effect of Zr:Sn ratio on the energy storage characteristics of lead lanthanum zirconate stannate titanate AFE ceramics having the nominal composition of (Pb_0.87_Ba_0.1_La_0.02_)(Zr_*x*_Sn_0.95 − *x*_Ti_0.05_)O_3_ (PBLZST, *x* = 0.6–0.8) [65]. The ceramics were fabricated through the conventional solid-state reaction technique. It was observed that the composition corresponding to *x* = 0.675 achieved a high value of saturation polarization of 43.5 μC/cm^2^ with a correspondingly large energy storage density of 2.05 J/cm^3^ and energy efficiency of 68.5% (1.4 J/cm^3^ of recoverable energy). This value was approximately seven times larger than other compositions reported within the same study. It was reported that when the Zr:Sn ratio increased from 0.625 to 0.675, especially from 0.65 to 0.675, the B sites are occupied by the Zr^4+^ ions of a larger radius with a noticeable increase of the unit cell volume and an enlargement of the B site ions’ activity space and relative displacement. This in turn leads to the increase of saturation polarization. In contrast to the above result, when the Zr content increased from 0.675 to 0.775, the unit cell volume increases, the increased unit cell volume is so small that the activity space and relative displacement of B site ions are decreased [65]. As a result, saturation polarization decreases with a further increase of Zr content. It was also observed that increasing the Sn content helped to lower the polarization back-switching energy which could help increase the energy efficiency. This study by Jiang *et al.* is an excellent example of how modification in the relative concentration of solid solutions can increase the energy density. A similar investigation was performed by Chen *et al.* who described the composition dependent energy storage performance in Pb_0.97_La_0.02_(Zr_*x*_Sn_0.95−*x*_Ti_0.05_)O_3_ (PLZST) (0.5 < *x* < 0.9) AFE thick films [67]. The films displayed tetragonal and orthogonal AFE phases, depending upon the chemical composition. The effect of Sn content and temperature on the dielectric losses was investigated and as the Sn content was increased, the electric field required for AFE to FE transformation decreased and the energy storage density increased. The maximum energy storage density of 5.6 J/cm^3^ (30 °C) and 4.7 J/cm^3^ (120 °C) with corresponding energy efficiency of 67% and 73% were obtained in Pb_0.97_La_0.02_(Zr_0.5_Sn_0.45_Ti_0.05_)O_3_ ceramic.

With respect to the same family of materials, Xu *et al.* reported the effect of Ba content on the stress sensitivity of AFE to FE phase transition in (Pb_0.94 − *x*_La_0.04_Ba_*x*_)[(Zr_0.60_Sn_0.40_)_0.84_Ti_0.16_]O_3_ ceramics [58]. Investigations were carried out by monitoring the electric field induced polarization and longitudinal strains under various compressive pre-stresses. The incorporation of Ba could significantly supress the stress sensitivity of the phase transition. It was also shown that the incorporation of Ba at the “A” site of the perovskite structure could reduce the transition field hysteresis; *i.e.*, the energy losses generated from repeated transitioning between AFE and FE states. This could further increase the energy and discharge efficiency. The addition of Ba disrupts large ferroelastic AFE domains, reduces the lattice distortion, and promotes the relaxor behavior; Ba therefore suppresses the ferroelastic contribution and leads to the phase transitions becoming insensitive to compressive stresses.

In a study by Ye *et al.* the modification of 0.80Bi_0.5_Na_0.5_TiO_3_-0.20Bi_0.5_K_0.5_TiO_3_ lead-free AFE ceramics has been examined by doping with SrTiO_3_ (ST) [66]. Although the study is reported as doping, the extremely large values (over 25 mol %) of incorporation could be aptly described as a solid-solution. The ceramics were prepared by a two-step sintering procedure and the effect of first sintering temperature and ST incorporation was investigated. The addition of 25 mol % ST could induce a double hysteresis loop (as in Figure 1d), which also significantly improved the energy storage characteristics with a maximum value of 0.84 J/cm^3^. The energy density could be further increased by optimizing the sintering temperature. It was observed that when the first sintering temperature is maintained at 1190 °C, the energy density could be further improved to a maximum value of 0.97 J/cm^3^. The incorporation of ST also led to an increase in the density of the sintered samples, which also improved the dielectric breakdown strength of the ceramics.

Wang *et al.* discussed the effect of Zr incorporation into the lead-free [(Bi_0.5_Na_0.5_)_0.94_Ba_0.06_]La_1−*x*_Zr_*x*_TiO_3_ AFE system [64]. Zr incorporation improved several ferroelectric attributes including polarization and energy density. A peak saturation polarization of 37.5 μC/cm^2^ was attained, while a peak energy density of 1.58 J/cm^3^ was observed for samples containing *x* = 0.02 Zr. The use of Zr doping helped to decrease both the remnant and saturation polarization which led to a reduced squareness of the hysteresis loops. It was noted that the slanted hysteresis loops thus obtained helped to increase the functional endurance of the ceramics against repeated application and removal of the actuating electric field.

The effect of Ba and Sr co-doping on the energy storage density of PLZST bulk ceramics having composition (Pb_0.85_Ba_0.08_Sr_0.03_La_0.03_)(Zr_0.74_Sn_0.22_Ti_0.04_) was studied by Wang *et al.* [63]. They reported that co-doping with Ba and Sr in a specific ratio could simultaneously lower the Curie temperature to 40 °C and transitioning hysteresis (Δ*E*) while also increasing the dielectric breakdown strength to 100 kV/cm; this resulted in a high energy density of 1.2 J/cm^3^ which was several times higher than the pristine composition. Co-doping was attempted to reduce squareness of the hysteresis loops and obtain slanted loops at a higher electric field. The effect created was similar to that demonstrated by glass incorporation (Figure 5e) to obtain slanted loops. However, no direct evidence of higher dielectric breakdown strength was provided and conclusions were only based on the slimness of the hysteresis loops, which could supposedly endure higher electric field.

Chemical modification in the form of doping and solid solutions has also been reported to influence the lattice parameters of AFE and field induced FE phase. This also has a direct bearing on the energy density of the concerned material. For example, in a study by Gao *et al.* investigations have been made with respect to the *c/a* (lattice parameters) ratio on the energy storage density on lead-free (0.9 − *x*)Bi_0.5_Na_0.5_TiO_3_-*x*BaTiO_3_-0.1K_0.5_Na_0.5_NbO_3_ (BNT-BT-KNN) bulk ceramics [103]. The samples were fabricated using conventional solid-state reaction technique having *x* = 0.060, 0.063, 0.066 and 0.069 compositions. X-ray diffraction was used to determine the lattice parameters of the sintered samples which was then correlated with the energy density obtained from *P*-*E* hysteresis measurements taken at an electric field of 5 kV/mm. It was observed that compositions having *x* = 0.063 had the largest *c*/*a* ratio of 0.70887 and had a correspondingly large polarization switching of 20.6 μC/cm^2^, with a maximum energy density of 0.424 J/cm^3^. It was reasoned that by adjusting the BNT/BT ratio in the given compositions, the cell parameters of the dominating tetragonal phase could be altered. This in turn would result in a greater tilting of the octahedra phase, thereby yielding an enhanced polarization switching.

Chen *et al.* attempted to fabricate slim-loop ferroelectric ceramics that were focussed for high-power pulse capacitor applications [104]. The researchers systematically investigated the effect of partial replacement of A and B site ions in Pb(Zr,Sn,Ti)O_3_ with Ba, La and Nb respectively. This was undertaken in order to reduce the hysteresis losses, remnant, and coercive parameters, respectively so that a high energy density could be obtained. The bulk ceramics were prepared by a conventional solid-state reaction technique. It was also observed that doping imparted excellent fatigue resistance as the stored charge was observed to degrade very slightly even after the application of 1000 cycles (from 230.49 to 192.96 μC). The PZST42/40/18 ceramics reportedly possessed a maximum energy density of 0.410J/cm^3^ at 6kV/mm while having a discharge efficiency of 76% after a thousand cycles.

Zhang *et al.* attempted to increase the energy density by fabricating (Pb_0.858_Ba_0.1_La_0.02_Y_0.008_)(Zr_0.65_Sn_0.3_Ti_0.05_)O_3_-(Pb_0.97_La_0.02_)(Zr_0.9_Sn_0.05_Ti_0.05_)O_3_ (PBLYZST-PLZST) AFE ceramics with varying phase ratios [68]. The variations were investigated with respect to structure, dielectric and energy storage characteristics of the ceramics. It was reported that with increasing PLZST content, the AFE temperature stability of the composites was improved. Composites with 50% PLZST displayed a considerable FE to AFE transition at an electric field of 130 kV/cm and a high polarization of 49.8 μC/cm^2^; this resulted in an overall energy storage density of 4.65 J/cm^3^.

In earlier work, Campbell *et al.* have attempted to improve the overall energy storage characteristics of fabricated device using a stacked multi-layer capacitor design for modified PbZrO_3_ AFE ceramics [105]. Both theoretical and experimental investigations were carried out with respect to DC measurements. An effective relative permittivity (dielectric constant) of ~4300 could be achieved and the device displayed a maximum energy storage density of 0.5 J/cm^3^. This was a rather non-conventional and hybrid approach where two different methods of multi-layer capacitor and chemical modification was used in conjunction to obtain higher energy density. Another non-conventional approach was recently reported by Guan *et al.* where they reported the fabrication and characterization of an AFE-like polymer for high energy density [106]. A poly(vinylidenefluoride-co-trifluoroethylene-co-chlorotrifluoroethylene)-graftpolystyrene (P(VDF-TrFE-CTFE)-g-PS) graft copolymer with 14 wt % PS side chains was synthesized using a solution growth method. The co-polymer was reported to possess “propeller-like” double hysteresis loops corresponding to AFE materials. However, in conventional PVDF and its various co-polymers, double loops have been reported to only exist at lower electric fields. In their work, Guan and co-workers claimed to confine PVDF lamellar crystals with a low polarizability PS interfacial layer. This could allow the AFE phase to be manifested at electric fields as high as 400 MV/m. The proposed method was also thought to be responsible for significantly reducing the hysteresis losses, thereby increasing the effective energy density. A maximum stored energy density of 20 J/cm^3^ was observed at an applied electric field of 500 MV/m. A similar approach was reported by Li *et al.* where PVDF co-polymers were grafted by poly(ethyl methacrylate) (PEMA) in a fashion similar to as described above [107]. The work was aimed at stabilizing the AFE phase at high poling fields (>500 MV/m). Grafting was reported to enhance the discharge energy density while lowering energy loss. The explanation provided by the authors stated that PEMA side chains were responsible for crystalline impediment and a polarization confinement effect. A highest energy density of 14 J/cm^3^ was reported at a discharge efficiency of 70% (550 MV/m) for sample containing 22 wt % PMA.

## 5. Influence of Other Factors

In addition to the discussions provided above, several other factors are also responsible for increasing the energy density in various morphologies of AFE ceramics. These include, but are not limited to, the effect of orientation, temperature and electric field. Hao *et al.* reported the energy storage performance of (100)-oriented Pb_0.97_La_0.02_(Zr_0.95_Ti_0.05_)O_3_ AFE films [62]. The films were prepared by sol-gel process and deposited on a Pt-buffered silicon substrate, having a thickness of approximately 1.7 μm. In this study it was highlighted that the effect of orientation could increase the saturation polarization. Additionally, it was reported that the stored energy density could be scaled with electric field to yield a maximum value of 12.4 J/cm^3^ at an electric field of 1120 kV/cm. Although it was not highlighted in the study, our observations also revealed that a significant reduction in hysteresis losses was observed when the temperature was raised from 20 to 100 °C, this is also expected to yield a larger energy storage density.

Ding *et al.* reported the enhancement of energy storage density in lead-free 0.89Bi_0.5_Na_0.5_TiO_3_-0.06BaTiO_3_-0.05K_0.5_Na_0.5_NbO_3_ ceramics [69]. This was achieved through a two-step sintering technique. The proposed method yielded a fine grain size and more uniform microstructure as opposed to those processed by single-step sintering. The maximum energy storage density obtained was 0.9 J/cm^3^, which was 300% larger than conventionally sintered ceramics. Zhang *et al.* have reported the beneficial effects of spark plasma sintering (SPS) technique for improving energy storage density in (Pb_0.858_Ba_0.1_La_0.02_Y_0.008_)(Zr_0.65_Sn_0.3_Ti_0.05_)O_3_-(Pb_0.97_La_0.02_)(Zr_0.9_Sn_0.05_Ti_0.05_)O_3_ (PBLYZST–PLZST) AFE ceramics [70]. It was reported that SPS is helpful to supress the diffusion behavior between the tetragonal and orthorhombic phases. Consequently, a high energy storage density of 6.4 J/cm^3^ was observed for a 50% PLZST sample with a material efficiency of 62.4%.

A unique study by Chen *et al.* attempted to elucidate the scaling behavior of energy density in Pb_0.99_Nb_0.02_[(Zr_0.60_Sn_0.40_)_0.95_Ti_0.05_]O_3_ AFE bulk ceramics [59]. Investigations were carried out with respect to electric field intensity and AC frequency of operation. The study was especially beneficial for providing scaling laws for relationships between applied electric field and stored energy density. It was observed that in low fields or high fields regions, the energy densities grow gradually with *E* increasing or *f* (frequency) decreasing. However, as *E* approaches a critical value the energy densities rise steeply with both *E* and *f*.

Zhang *et al.* attempted to illustrate the charge-discharge properties of La-modified Pb(Zr, Sn, Ti)O_3_ ceramic capacitors as a function of varying electric fields [89]. Direct measurements were made for pulse discharge current-time curves under different electric fields. Upon increasing the electric field from 3 to 3.5 kV/mm, large increments in polarization and discharge current were obtained. Under an electric field of 4 kV/mm, the first current peak was found to surpass 1.1 kA, and more than 80% electric charge stored can be released in <65 ns. In addition, the fabricated ceramics were reported to withstand 2000 charge-discharge cycling with no significant degradation of properties.

## 6. Disadvantages Associated with Anti-Ferroelectric Materials

Despite their numerous advantages and superior performance there are a few factors which serve to limit their practical application as energy storage materials. The foremost among them is the large piezoelectric noise. Piezoelectric noise is the unwanted field-driven actuation of a piezoelectric material. When subjected to large electric field intensity AFE materials respond with an AFE to FE phase transformation which is accompanied by a large strain response. For AFE materials this actuation generally results in a strong unwanted vibration which creates the requirement for damping or isolation lest it begin to harm the structure altogether. Hence, one of the biggest concerns in this field has been to curb or reduce the piezoelectric response, especially for applications requiring alternating current voltages.

Another major problem associated with use of AFE materials for energy storage application is hysteresis and performance degradation. Like all ferroelectric materials, AFEs also suffer from polarization fatigue wherein the magnitude of saturation polarization decreases with increasing number of electric cycles. Also known as functional fatigue, it could also simultaneously result in lower dielectric constant and piezoelectric response with a gradual increase in remnant polarization and dielectric loss. Unlike FE materials the fatigue behavior in AFE materials is rather less explored and proper theories exist which can accurately model the onset or advancement of fatigue in AFE ceramics. But despite all their disadvantages, it has been often reported that the fatigue performance of AFE materials is, in general, better than most FE materials. Nevertheless, if these two main disadvantages could be overcome, it would seriously boost the prospects for the development of a practical AFE based capacitor.

## 7. Concluding Remarks

As modern electronic devices aim for simultaneous miniaturization of size and increase in performance, the requirements for high energy density capacitors will continue to grow. It has been argued that among the potential solid dielectric materials, anti-ferroelectric materials possess the best combination of properties for the development of solid-state capacitors for future electronic applications among other areas. The interest in AFE materials stems from the lack of hysteresis (coercive field, remnant polarization, dielectric losses), while also possessing high energy storage density and discharge efficiency. However, despite evident advantages, little has been done to fully harness the potential of AFE materials. In this paper, we have presented a timely review of the important technological advancements that have been made in this field to date. This has been primarily aimed at inspiring interest towards research in the field of energy storage through the use of AFE materials. Discussion has been provided for the origin of anti-ferroelectricity in bulk materials and how this is best employed for energy storage applications. This was followed by discussion of various physical and chemical parameters that can be used for enhancing and tuning the energy storage density in bulk and thick film AFE materials. The foremost physical methods include the use of mechanical confinement (compressive pre-stress) and self-confinement while the primary chemical techniques employ glass additions and doping. After a thorough review of the literature present in this area along with the contemporary advancements recently achieved, the authors are of the opinion that AFE materials possess significant potential for the development efficient solid state capacitors. We have only uncovered a small fraction of the same and rigorous research is needed to expedite successful commercial ventures.

## 8. Challenges and Future Prospects

Despite their evident advantages, much effort is still required to successfully bridge the gap between laboratory demonstrations and large scale commercial production of AFE ceramic capacitors. There remains a lack of suitable lead-free AFE material which can perform to a similar level of the best lead-based AFE ceramics. In the remarkable progress towards the development of lead-free AFE materials, recent work by Shimizu *et al.* deserve special attention [71]. As is evident from this review, and as other excellent reviews reported in the literature [3,50,53,80,94], there is a need to develop suitable AFE materials. The majority of materials which display useful AFE characteristics are lead-based while the few lead-free compositions perform relatively poorly. Shimizu and co-workers have proposed a novel family of AFE systems which could be potentially employed for various applications including pulse power application and power electronics. These ceramics belong to the nominal composition of *x*CaZrO_3_-(1−*x*)NaNbO_3_ where (0 ≤ *x* ≤ 0.10) and the AFE characteristics can be stabilized in NaNbO_3_ by lowering the tolerance factor. This was achieved by simultaneous substitution of Zr and Ca ions in place of Na and Nb sites respectively. This helps lower the polarizability and tolerance factor in the solid solution while maintaining charge neutrality. Transmission electron microscopy analysis revealed that in samples containing *x* = 4 and 5, only AFE domains were observed as opposed to pure (undoped) ceramic which displayed a mixture of AFE and FE phases, at room temperature. Samples having 0.02 ≤ *x* ≤ 0.05 exhibited double hysteresis loops, indicating AFE behavior. The study was also supported by first principle analysis and upon subsequent investigation by our group, we observed that the ceramics possessed an impressive energy storage density of 0.91 J/cm^3^. These are very promising results and they have pointed towards new fields of investigations which will help us to expand the AFE materials currently available for storage applications.

**Figure 6:**
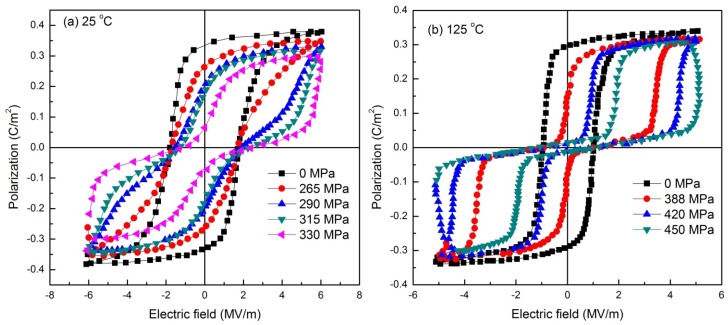
*P*-*E* hysteresis loops of the Nb modified 95/5 PbZrTiO_3_ bulk ceramics, obtained as a function of hydrostatic pressure at the temperatures of (**a**) 25 °C; (**b**) 125 °C. Data adapted from [108].

**Figure 7:**
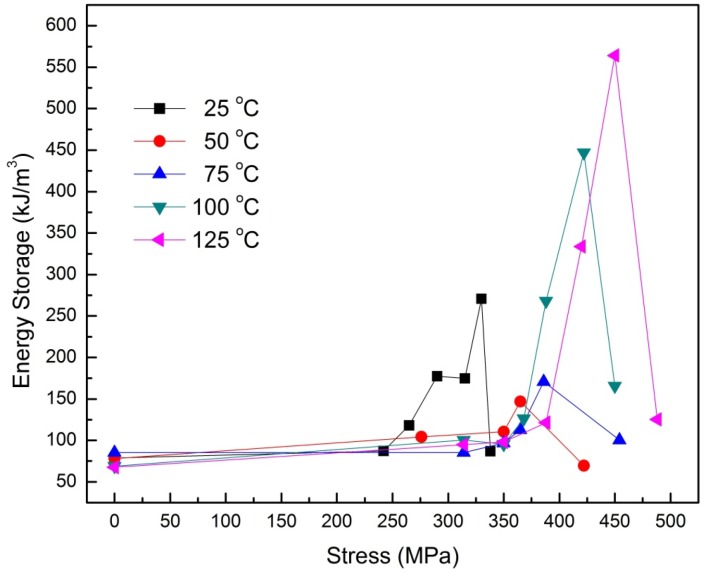
Energy storage density of Nb modified 95/5 PbZrTiO_3_ bulk ceramics as a function of operating temperature and applied hydrostatic pressure. Data adapted from [108].

In recent work, Dong *et al.* reported the pressure, temperature, and electric field dependence of phase transformations in Nb modified 95/5 PbZrTiO_3_ bulk ceramics [108]. Here, the bulk ceramics were subjected to electric field hysteresis under the application of varying temperature and applied hydrostatic pressure. An interesting observation was that under a sufficiently high pressure (>300 MPa at 25 °C) a FE to AFE phase transformation can be induced. The double hysteresis loops became more pronounced on increasing the pressure up to 500 MPa while any significant increase in temperature or electric field was observed to hinder the formation of this pressure induced AFE phase. Figure 6 shows the *P*-*E* hysteresis loops of the bulk ceramics, obtained as a function of hydrostatic pressure at the temperatures of 25 and 125 °C. In addition, Figure 7 shows the energy storage density of these ceramics as a function of stress and temperature. A maximum energy density of 0.55 J/cm^3^ was obtained at a temperature of 125 °C for an applied pressure of 450 MPa. The study holds promising results and must be extended to other compositions to see if there is potential to improve the energy storage density in such ceramics.

With the global initiative to phase out lead from ferroelectric and anti-ferroelectric ceramics for technological applications, efforts are required to develop and fabricate new lead-free materials with the required dielectric and AFE properties. Furthermore, extensive research is also needed in order to develop new and economic methods for processing and fabrication of AFE materials which will help to improve their energy storage characteristics. Finally, the AFE to FE transformation is accompanied by large volumetric strain response which is often unwanted behavior and termed as “piezoelectric noise” [109,110]. This can lead to mechanical failure, degradation, and reduces the functional and mechanical endurance of AFE ceramics. Efforts are also required to reduce or remove this unwanted field driven strain response so that the practical appeal of such ceramics can be enhanced. AFE materials have significant untapped potential as high energy and power density solid-state ceramics and present ample scope of improvement.
